# A Qualitative Evaluation of the eaTracker^®^ Mobile App

**DOI:** 10.3390/nu10101462

**Published:** 2018-10-09

**Authors:** Jessica R. L. Lieffers, Renata F. Valaitis, Tessy George, Mark Wilson, Janice Macdonald, Rhona M. Hanning

**Affiliations:** 1 College of Pharmacy and Nutrition, University of Saskatchewan, Saskatoon, SK S7N 2Z4, Canada; jessica.lieffers@usask.ca; 2 School of Public Health and Health Systems, University of Waterloo, Waterloo, ON N2L 3G1, Canada; rfvalait@uwaterloo.ca; 3 Hospital for Sick Children, Toronto, ON M5G 1X8, Canada; t.george@mail.utoronto.ca; 4 Dietitians of Canada, Toronto, ON M5R 1C1, Canada; mark@wilson5.ca (M.W.); janice@childhoodobesityfoundation.ca (J.M.); 5 Childhood Obesity Foundation, Vancouver, BC V5Z 1M9, Canada

**Keywords:** mobile applications, adults, nutritional science, qualitative research

## Abstract

Background: eaTracker^®^ is Dietitians of Canada’s online nutrition/activity self-monitoring tool accessible via website and mobile app. The purpose of this research was to evaluate the eaTracker^®^ mobile app based on user perspectives. Methods: One-on-one semi-structured interviews were conducted with adult eaTracker^®^ mobile app users who had used the app for ≥ 1 week within the past 90 days. Participants (*n* = 26; 89% female, 73% 18–50 years) were recruited via email. Interview transcripts were coded using first level coding and pattern coding, where first level codes were grouped according to common themes. Results: Participants mentioned several positive aspects of the mobile app which included: (a) Dashboard displays; (b) backed by dietitians; (c) convenience and ease of use; (d) portion size entry; (e) inclusion of food and physical activity recording; and (f) ability to access more comprehensive information via the eaTracker^®^ website. Challenges with the mobile app included: (a) Search feature; (b) limited food database; (c) differences in mobile app versus website; and (d) inability to customize dashboard displayed information. Suggestions were provided to enhance the app. Conclusion: This evaluation provides useful information to improve the eaTracker^®^ mobile app and also for those looking to develop apps to facilitate positive nutrition/physical activity behavior change.

## 1. Introduction

Over the past decade, mobile devices and their applications (“apps”) have become an integral part of the everyday lives of many Canadians. The Canadian Radio-television and Telecommunications Commission reported that in 2016, 87%, 77%, and 54% of Canadian adults owned cellphones, smartphones, and tablets, respectively, which is up from 80%, 51%, and 26%, respectively, in 2012 [1]. Use of mobile apps for health-related purposes has also become popular amongst the general public. A recent survey study of mobile phone owners from the United States found that almost 60% of respondents had downloaded a mobile app with health-related content; the authors also found that use of apps for nutrition and fitness was common [2]. In addition, some apps for monitoring eating and activity behaviors (e.g., MyFitnessPal (Under Armour Inc., Baltimore, MD, USA)) have had millions of downloads. The availability of health apps is also expanding daily—a recent report found that about 200 new health apps are added each day [3]. Dietitians are also now commonly encountering clients interested in using nutrition apps in their practice [4,5]. However, despite the popularity of nutrition (and activity) apps, many may have been developed without visible health professional input [6].

Dietitians of Canada’s eaTracker^®^ (http://www.eatracker.ca/) is a free, publicly available bilingual (English and French) web-based tool that allows members of the public to track their eating and/or physical activity behaviors and compare them to recommendations (including those set by Health Canada). Users create an account and enter demographic information including their month and year of birth, sex, height, weight, self-reported activity level, pregnancy/breastfeeding status, postal code, province of residence, and country of residence, which allows personalized recommendations to be determined. Users then have the ability to enter eating and physical activity behaviors via a database of available choices (~4500 food items from the Canadian Nutrient File (version 2010) which contains information on average nutrient values for foods available in Canada [7], and ~159 activities). Following data entry, users are able to receive feedback on consumption of calories, Canada’s Food Guide [8] servings, 22 nutrients as well as information on physical activity including minutes of activity, minutes of low effort, moderate effort, high effort, and muscle and bone strengthening exercise, and number of calories burned through exercise. eaTracker^®^ also contains other tools such as a recipe analyzer (which allows users to enter ingredients to obtain a nutritional analysis of their recipe, and save this recipe to expedite future entry of this recipe into eaTracker^®^), a goal setting and tracking tool (My Goals) (described elsewhere) [9,10], and the ability for a dietitian coach to view intake and activity patterns of a group of clients, and provide comments.

**Figure 1:**
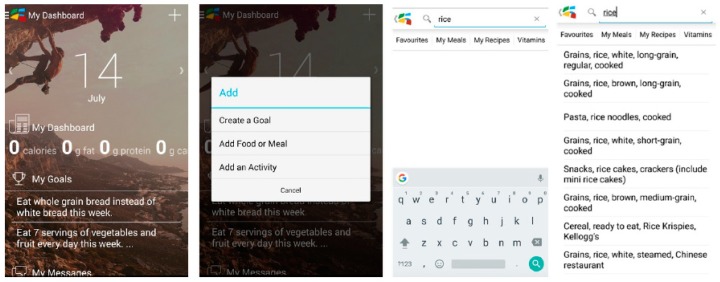
eaTracker^®^ mobile app screenshots.

In 2014, Dietitians of Canada released free iOS™ and Android™ eaTracker^®^ mobile apps available via the Apple App Store^®^ (Cupertino, CA, USA), and Google Play™ store (Mountain View, CA, USA). The mobile app can be used either by itself or in conjunction with the eaTracker^®^ website. The mobile app allows users to create/access their account, to log and receive feedback on eating and physical activity behaviors, and to set and track goals using the My Goals feature. Users are able to receive feedback on intakes of energy, macronutrients, and number of servings from the four Canada’s Food Guide food groups as well as activity behaviors via the mobile app. Users also have the option to visit the eaTracker^®^ website to obtain the more comprehensive assessment of their eating and activity behaviors and use the recipe analyzer. Screenshots of the eaTracker^®^ mobile app are shown in Figure 1.

The purpose of this research was to conduct an evaluation of the eaTracker^®^ mobile app using qualitative one-on-one semi-structured interviews with users. This evaluation will support future modifications to the eaTracker^®^ mobile app and the development of other credible, user-friendly, and effective nutrition and physical activity behavior change mobile apps to optimize the nutritional status of Canadians.

## 2. Materials and Methods

An advisory committee (*n* = 5 Dietitians of Canada staff members; researchers from University of Waterloo) oversaw the project design, methods (including interview protocol), and analyses. The Dietitians of Canada information technology team also provided expertise and support. The University of Waterloo Office of Research Ethics provided ethics approval (ORE: #20671; approval date: April 30, 2015). The Consolidated Criteria for Reporting Qualitative Research (COREQ) checklist [11] guided study reporting.

Approximately 30 participants were desired a priori for this study; a convenience sampling strategy was used. eaTracker^®^ users were approached about the study through an email invite sent via FluidSurveys (FluidSurveys, Ottawa, ON, Canada). Dietitians of Canada sent an email invite on two occasions (May 12, 2015; June 15, 2015) to eaTracker^®^ users who: (a) Were ≥18 years, (b) Southern Ontario residents (based on self-reported postal code in their eaTracker^®^ account), (c) had used eaTracker^®^ within the past 90 days and, and (d) previously provided permission to be contacted by Dietitians of Canada. The email invite provided a link to an online survey where users were asked whether they had specifically used the eaTracker^®^ mobile app within the past 90 days; if they responded “yes”, they were invited to leave their name and contact information. A research assistant (TG) then contacted individuals via email or phone to provide more information about the study. Interested users who had used the eaTracker^®^ mobile app for ≥1 week were invited to complete the interview.

One-on-one, semi-structured interviews were conducted by a TG, a female research assistant who had recently completed a Bachelors degree in Health Studies (with a minor in Nutrition) and was trained in qualitative research methods. The researcher who conducted the interviews was not using eaTracker^®^ at the time of the interview, and was at an arms-length from Dietitians of Canada in order to prevent bias. Participants did not know anything about the researchers except information provided via the information letter and consent forms. Participants were told that the purpose of the study was to evaluate the eaTracker^®^ mobile app by obtaining information on user perspectives regarding app content, service, and functionality. No relationships with participants were established prior to study commencement. Interviews were conducted in-person (in public locations e.g., coffee shops), by telephone, or Skype™ (Microsoft Corporation, Redmond, WA, USA). Interviews were conducted at a time that was convenient for both individuals, and no other individuals were present at the interview except the participant and researcher. Several interviews were conducted by telephone, as participants were located across Southern Ontario; however, if participants were located within close proximity to researchers, efforts were made to conduct in-person interviews. All participants provided written (in-person interviews) or verbal (telephone or online interviews) informed consent. The semi-structured interviews were guided by an interview protocol with open-ended questions designed to address study objectives (Supplementary S1). Both clarifying and elaborating probes were used to gather additional data [12]. The interview protocol was pilot tested with two individuals from the target population prior to data collection. Field notes were taken during interviews. Participants were provided with a free Dietitians of Canada cookbook following the interview as a thank you gift. No participants dropped out after completing the interview. Interviews were completed until data saturation was achieved; no repeat interviews were conducted. All interviews took place between May 2015 and August 2015.

All interviews were audio-recorded and transcribed verbatim. Detailed notes were taken for participants who did not consent to the audio-recording. Any identifying information in the transcripts was removed to maintain confidentiality. Transcripts were reviewed to correct any errors; transcripts were not returned to participants for comment and/or correction, and participants did not provide feedback on study findings.

Data were analyzed using content analysis [13,14]. The interviews were coded by a single trained coder (TG) using NVivo 10 for Mac (QSR International Pty Ltd., Doncaster, Australia). First level coding and pattern coding were used, where first level codes were grouped according to common themes which were derived from the data [15]. To ensure reliability of coding, a second experienced coder reviewed a subset of transcripts (~10%) and associated codes, and themes. Any disagreements were discussed until consensus was reached [16].

## 3. Results

In total, *n* = 4135 and *n* = 1082 eaTracker^®^ users were sent an email message on May 12, 2015 and June 15, 2015, respectively inviting them to participate in the study. In total, *n* = 129 users completed information on the recruitment survey. Of those, *n* = 67 eaTracker^®^ users who had used the mobile app in the past 90 days provided contact information and were contacted by TG, and in total, *n* = 26 users participated in the one-on-one semi-structured interview (average length: 36 min; range: 20–64 min); one interview was not recorded because of participant request (detailed notes were taken instead for this interview).

Table 1 shows participant demographics and interview methods. Overall, 88.5% of participants were female, and 73.1% were 18–50 years of age; just under 60% of interviews were done by phone. The distribution of age and sex for study participants generally reflects the overall population of eaTracker^®^ users in Canada from July 5, 2015 to September 2, 2015 (*n* = 2265) (79.8% female; 75.7% 18–50 years).

Participants reported various nutrition and physical activity goals including: Improving specific eating habits (e.g., follow food guide, consume a balanced diet, plenty of vegetables, decrease saturated fat intake, reduce sodium intake, decrease meat intake, decrease intake of “bad foods,” meet iron requirements) (*n* = 21 participants), weight management (*n* = 9), and attaining recommended activity levels (*n* = 9).

In total, *n* = 18 and *n* = 11 participants were using the iOS™ and Android™ eaTracker^®^ mobile apps, respectively. Participants used various devices to access the app; in total, *n* = 13, *n* = 9, *n* = 5, and *n* = 2 participants accessed the mobile app via an iPhone^®^, Android™ phone, iPad^®^ tablet, and BlackBerry^®^ phone, respectively. Participants mentioned finding out about the eaTracker^®^ mobile app through different channels; the most common ways were via the eaTracker^®^ website, app stores, and through their dietitian or a dietitian they followed on social media. Some participants were also informed about the mobile app through school, the EatRight Ontario website (now rebranded as http://www.unlockfood.ca/), family and friends, trainers, and media.

Information on duration of eaTracker^®^ mobile app use was obtained for *n* = 23 participants. In total, *n* = 9 participants had used the eaTracker^®^ mobile app between one and three weeks, *n* = 4 for about two months, *n* = 7 between three and six months, and *n* = 3 for a year. Most participants described themselves as ‘daily users’ which could include using the app multiple times/day, using the app after every meal, or using the app at specific times (e.g., morning to enter foods eaten the day before). In addition, *n* = 5 users mentioned that they used the app on a weekly basis, and *n* = 4 used the app less often than weekly (e.g., every few months). Of the *n* = 26 participants, *n* = 13 said that they would continue to use the eaTracker^®^ mobile app in the future, *n* = 8 said that they would not continue to use the mobile app, with the remaining being unsure if they would continue or discontinue eaTracker^®^ mobile app use. Reasons for discontinuation varied and included, for example, preference for other apps, and the food search feature being difficult to use.

In total, *n* = 10 participants mentioned using the eaTracker^®^ mobile app at home. Participant 011 stated, “If I’m out for a meal, I’ll do it when I get home; always at home. It just takes too long to do it when I’m out … you have to type in the whole word.” However, many participants also reported using the mobile app in any setting and even found that it could be “a discussion point.” Typically, these participants used the mobile app as soon as they consumed food or beverages. A few participants also mentioned that the mobile app helped them to increase awareness of the foods they were eating throughout the day. Participant 026 mentioned that the use of the mobile app allowed her to “… see where I was adding the most calories, cause a lot of these calories were hidden to me. Once I put them in eaTracker I noticed where I was adding too much.”

### 3.1. Positive Aspects of the eaTracker^®^ Mobile App

Participants mentioned several positive aspects of the eaTracker^®^ mobile app which included dashboard displays, backing by dietitians, convenience and ease of use, portion size entry, and inclusion of both food and activity recording components. These findings are described in detail below.

#### 3.1.1. Dashboard Displays

The dashboard display’s information about quantities of calories (kcal), macronutrients (g), Canada’s Food Guide servings consumed as well as goals set and physical activity behaviors (screenshot in Figure 1). Participants felt positively about how the information was organized on the dashboard and found it visually appealing. Participant 020 mentioned, “I do like the dashboard format where it’s like a summary at a glance.” Participant 006 mentioned, “I like that (the dashboard is) not too overly physical, like there’s not too many graphics or pictures and all that, things that typically take longer to load too that I don’t need.” Participants also liked other aspects of the dashboard including the ability to change the background picture and to use a swiping motion to view information on behaviors logged for previous days.

#### 3.1.2. App Backed by Dietitians

Several participants liked that the mobile app was developed by a reputable organization (Dietitians of Canada), backed by dietitians, and contained Canadian content. Some participants reported trusting the validity and accuracy of the information presented in the mobile app. For example, Participant 006 mentioned: “… the Canadian focus of it too-right? There’s a lot of US based stuff; I wanted something that was in Canadian metrics and Canadian context.” The fact that Dietitians of Canada developed the mobile app was a motivating factor to continue use for some participants such as Participant 016: “I don’t know I guess I just haven’t given up on it yet. Because it’s gotta be worth it if the Dietitians of Canada suggest it.”

#### 3.1.3. Convenience and Ease of Use

Most participants found the mobile app was convenient, easy to use, and an easy way to record both their eating and activity behaviors. Participants also described the mobile app as convenient because having it on their mobile devices allowed them to record their eating and physical activity behaviors during leisure time or soon after they had a meal. The perspective that mobile apps are easier to access versus computers was also described by several participants. Participant 018 explained, “it’s a lot easier to pick up a phone like off a counter than if you go onto a computer and login and everything.” In addition, participants that used the website and the mobile app together enjoyed the flexibility of being able to access their eaTracker^®^ account in different ways and felt that the two methods to access the tool complimented one another. Participant 001 explained, “I like how they’re integrated, (when I) input something on the mobile app, I don’t have to go back to the website to change it or make sure it’s correct and put something on the website.”

#### 3.1.4. Portion Size Entry

The eaTracker^®^ mobile app provides users with several options for entering portion size information for the foods they consume (e.g., volume, weight, and count-based units). Participants felt positively about having access to several units to enter food portion sizes, and felt this was unique to this mobile app. Participant 008 stated, “I like all the quantities that they provide, that’s really helpful.” This participant also went on to compare the number of measurements provided in the eaTracker^®^ mobile app vs. other non-Canadian commercial apps. They explained, “(name of other commercial app) do(es) have that as well but sometimes they don’t have (the units) that you want and I found that (the eaTracker^®^ mobile app) was actually better.”

#### 3.1.5. Includes Both Food and Physical Activity Recording

A couple of participants liked that the mobile app included both food and activity recording components. They felt positively about the fact that the activity and food entry were separate distinct components with similar layouts all housed within the same mobile app. Participant 022 stated, "I like that you can enter your physical activity as well as your, your food in there because you know they both go hand in hand when you’re worrying about your health.” Even participants like Participant 001 who had never reported using the physical activity feature appreciated that this functionality was available. They stated, “(what) I like about the app is that you can add how much physical activity you’ve done, I’ve never used that in the app, but you could, so I like that cause with a lot of the other apps it’s strictly a food app or strictly health.”

#### 3.1.6. Ability to Access More Comprehensive Information via the eaTracker^®^ Website

A few participants also liked that they could visit the eaTracker^®^ website to retrieve a “more detailed description” of their food intake through nutritional reports that were not available via the mobile app. Participant 005 explained that “(the website) gave more information in terms of what vitamins, the nutritional concepts. (Other mobile apps are) strictly limited to calories, fats, sodium and a hand full of nutrients but the eaTracker app was more accurate in terms of stuff you wouldn’t regularly think about.”

### 3.2. Challenges with the eaTracker^®^ Mobile App

Participants mentioned some challenges with the eaTracker^®^ mobile app which included difficulties with the search feature, limited food database, differences between the mobile app and the website, and inability to customize nutrition variables displayed on the mobile app dashboard; these four findings will be discussed in more detail. Participants also mentioned other challenges, which included having lunch as the default meal, inability to use the mobile app without Wi-Fi or cellular data, and finding the mobile app background distracting.

#### 3.2.1. Search Feature

Over half of participants described various difficulties with the eaTracker^®^ food search feature which made the mobile app tedious to use. There were also a few concerns mentioned with the activity search feature, although these comments were less common. One reported difficulty was that the search did not bring up relevant items. Participant 025 explained the challenge: “After I started using it, I found it really almost too detailed … like when I was looking for like a thing I have eaten, I got 50 options that came up. But I can’t scroll through and I wasn’t able to find a way to filter them in order to find what I was looking for-something that was close enough.” Another challenge was that the search provided unrelated items. Participants who had used other mobile apps for food data entry also discussed the benefit of entering foods using a barcode scanner; participants described this entry as quick as it did not require extra time to search for individual foods. Another participant provided a suggestion that the mobile app bring up common foods eaten at the same time each day when the app is opened (instead of having to search for foods) to simplify data entry.

#### 3.2.2. Limited Food Database

Another concern was the limited food database. Almost 50% of participants had concerns about missing food items from the database, some of which they believed to be common, such as Greek yogurt. Participant 006 mentioned, “it’s limited, they don’t have Greek yogurt for instance.” Participant 020 also mentioned: “I didn’t feel like the database of food and nutritional information had represented what I had actually eaten.” Participants often compared the eaTracker^®^ mobile app food database to other commercial mobile apps which have much larger food databases, which, according to participants, contained everything from restaurant foods to packaged foods, and trendy foods (e.g., gluten free foods, Goji berries).

Several participants suggested efforts should be made to improve the food database. Participant 021 mentioned: “I would say that it would be good to work on the database of the food. That would be my biggest comment-just to have more foods available to input would be perfect.” Participants suggested different ways to improve the food database. One suggestion was to add more restaurant foods. Participant 003 mentioned: “having restaurants-that would be handy, including Canadian restaurants, because nowadays a lot of people are eating out at Tim Hortons or Starbucks or whatever …” Another suggestion was allowing users to update the database on their own. Participant 025 suggested, “I wonder if there might be an opportunity instead of looking for an option in the list that would represent what you just had-if you could take a picture or do something with the nutritional label which is just standard and input it saying this is what I had, right?” Another suggestion was having a community-based database where users could add in nutrition information from the products they typically consume which would allow for a greater number of options to be available for users.

#### 3.2.3. Mobile App Differs from the Website

While most participants felt the eaTracker^®^ mobile app was simple to use, some participants who used the eaTracker^®^ website previously did not enjoy the layout and interface of the mobile app because it did not resemble the website. Participant 012 mentioned: “I didn’t really understand (the eaTracker^®^ mobile app). It was just the interface; I wasn’t used to it cause it did look different from the website … so yeah, but I definitely didn’t start using it immediately.” In addition, some participants mentioned concerns that some feedback information available on the website was not available via the mobile app.

Over half of participants wanted more eaTracker^®^ website features available via the mobile app. Several participants wanted to be able to access progress graphs available on the eaTracker^®^ website via the mobile app. Participant 012 mentioned: “Well, personally, I would definitely like to see graphs of everything, so everything that I can see on the website, I should be able to see on eaTracker mobile app in terms of that analysis portion-so both the like micronutrients, macros as well as the like food groups.” Others discussed wanting to be able to access the recipe analyzer via the mobile app. Participant 022, suggested, “I would like to see the recipe analyzer feature added to the app, maybe that’s way too complicated and there’s a reason why they haven’t put it on there but I would certainly use it.”

#### 3.2.4. Inability to Customize Dashboard Displayed Information

As mentioned previously, participants liked the concept of the dashboard. However, a few participants mentioned a limitation of the eaTracker^®^ mobile app was the inability to customize the specific nutritional variables displayed. Participant 022 mentioned, “I would like to see them add sugar to that-like grams of sugar, to that little part that goes across it (i.e., dashboard) … I think a lot of people are watching their sugar these days and it would be handy to know how much sugar is in the foods that you’re eating.” Participants had varied preferences on the variables that they wanted to be included or excluded depending on their dietary pattern (e.g., vegetarian) or disease state (e.g., diabetes) and wanted the app to be able to accommodate those preferences.

Participants also suggested making the mobile app more interactive by providing customized recommendations based on user-entered data (e.g., goals, nutrient intake, common foods). Participant 008 stated, “if I could just click on a button, say on the day, when I’m low on something I hit a button and it tells you to ‘try eating this’ and choose a snack that had more of that in it.”

## 4. Discussion

eaTracker^®^ is one of only a few Canadian mobile apps to support nutrition (and activity) behavior change. The current evaluation highlights the enthusiasm of users and provides rich feedback to enhance this mobile app as well as other electronic health tools to optimize nutrition (and activity) behaviors to prevent and manage chronic diseases such as cardiovascular disease, diabetes, and cancer.

One key finding from this study was that participants liked that the mobile app was developed by a reputable organization and for some participants, this was a motivating factor to continue use. Concerns about mobile app credibility and accuracy were also mentioned in a related study by Dennison et al. [17]. This finding is important as many health, nutrition, and weight management apps are not developed with input from health care professionals and professional organizations; Nikolaou and Lean [6] recently found that <1% of weight management apps were developed with visible professional input. Professionals and professional organizations should consider becoming involved in app development and making this involvement clearly visible to users. In addition, implementation of easy ways for consumers to identify nutrition apps developed by dietitians (and other reputable professionals or organizations) may be helpful. One strategy may be to have a list of apps and a badge for those developed with this type of input, which is similar to what Dietitians of Canada does to identify blogs written by dietitians (Dietitians of Canada Member Blogs) [18].

Similar to other qualitative data on user experiences with nutrition mobile apps [17,19,20,21,22,23], this study found that food data entry is a key topic that affects satisfaction with these types of tools. Previous qualitative studies with related mobile apps have also identified that users have a strong desire to record the foods eaten as precisely as possible [17,23], which may be a reason for the high frequency of this type of comment among participants in the current study. Participants in this study liked that multiple units were available for food data entry with the eaTracker^®^ mobile app (which is a limitation of many other similar mobile apps) [19]; however, they encountered challenges with the search feature, as well as frustrations with the small food database. Generally, other studies have found that large databases are convenient and well-liked because of the large variety of foods available which allows an exact item to be found [19,21]. However, difficulties finding correct foods in large databases have also been reported [19,20]. Difficulties with both large and small food databases suggests that issues with food databases are present regardless of database size. While the eaTracker^®^ food database is smaller and does not offer the ability to self-enter foods compared to databases used in other publicly available apps, it has the potential to provide feedback on a larger selection of dietary variables because the Canadian Nutrient File database is used rather than relying on information only available on food labels. Barcode scanners to streamline data entry have been previously reported to be well-liked [19,23]; however, they are only useful for entry of packaged foods and errors with these tools have been reported [19]. Future modifications to eaTracker^®^ and other mobile apps will need to weigh the pros and cons of different options for food entry. Additionally, when the method(s) are chosen, it is important that users are educated on the rationale and pros and cons of the chosen food entry option and strategies to ensure success with data entry for the chosen option. For example, if smaller databases are chosen to be used, users should be provided with strategies on how to find the correct foods (e.g., education on searching for the type of food instead of the brand name, e.g., searching for hazelnut spread instead of Nutella^®^) to help promote success and satisfaction with the tool. In addition, regardless of database size, implementing strategies to streamline data entry (e.g., via favorites, commonly entered items, recently entered items, optimizing the search feature) would be a worthwhile endeavor.

A notable finding from this study was that participants who had been previous users of the eaTracker^®^ website initially found the eaTracker^®^ mobile app difficult to use. To our knowledge, this is the first time that this qualitative finding has been reported in users of a mobile nutrition (and activity) self-monitoring tool. This finding suggests that users may find transitioning to a different format to be challenging and that it may be necessary to implement strategies to help improve their success.

Participants mentioned that one of their most liked features of eaTracker^®^ is the in-depth feedback provided on several dietary variables; this feedback is provided in different ways including charts and graphs. Some participants wanted mobile app access to include more of this personalized information currently available only on the eaTracker^®^ website. This finding aligns with previous studies in this area which have found that having access to numbers and graphs about progress in a nutrition and/or physical activity behavior change mobile app is generally well-liked and can be motivational [21,23,24,25,26,27]. In addition to having the ability to personalize which eaTracker^®^ feedback information is displayed on the mobile app, personalization of other aspects of the app (e.g., automated recall of favorite foods) (as has also been found in other related studies [28]) is also desired. This should be considered in future eaTracker^®^ mobile app modifications and development of future apps.

### Strengths and Limitations

This study has several strengths. Sampling occurred until data saturation was reached. In addition, a variety of participant types were chosen without exclusively focusing on individuals using the app for weight management. In addition, we captured information on real-world experiences as opposed to experiences of use as part of a research trial.

While this study has several strengths, there are some limitations that should be mentioned. It should be noted that only a small subset of eaTracker^®^ users responded to the email invitation which is common for these types of invitations. In addition, participants who completed the interview may be more motivated and willing to contribute feedback compared to the general user group. Participants were also primarily female and 18–50 years of age; however, this distribution generally reflects the overall population of eaTracker^®^ users. In addition, information on education level, income, and ethnicity was not collected from participants.

## 5. Conclusions

This evaluation of the eaTracker^®^ mobile app provides important insight on real-world user experiences with mobile apps for nutrition (and activity) behavior change. Users liked that the app was developed by a reputable organization, and had multiple ways to enter food data. Professionals and developers should keep in mind that users may have difficulty transitioning between a website and mobile app, and that finding ways to streamline data entry should be a priority. In addition, allowing users to personalize mobile apps would likely help to increase satisfaction. Ultimately, higher user satisfaction may result in improved app adherence which may help to improve nutrition (and activity) behaviors to decrease the burden of chronic disease.

## Figures and Tables

**Table 1:** Participant demographics and interview method.

	Number (%)
Sex	
Female	23 (88.5)
Male	3 (11.5)
Age (years)	
18–30	9 (34.6)
31–50	10 (38.5)
51–70	7 (26.9)
Interview Method	
Phone	15 (57.7)
Online (e.g., Skype)	2 (7.7)
In-Person	9 (34.6)

## Supplementary Materials

The following are available online at http://www.mdpi.com/2072-6643/10/10/1462/s1, S1: Interview Protocol for eaTracker^®^ Mobile App Users.
