# Supplementary material for: A Qualitative Evaluation of the eaTracker® Mobile App
Source: Nutrients. 2018 Oct 9;10(10):1462. doi: 10.3390/nu10101462 (PMC6213652; doi:10.3390/nu10101462)
Supplement: Supplementary file 1 [file nutrients-10-01462-s001.zip › nutrients-356411-supplementary.docx]

**Interview Protocol for eaTracker® Mobile App Users**

Anonymous Interviewee ID:

Interviewer:

Date of Interview:

Time of Interview:

Interview method (circle): In person Phone Online (Skype/FaceTime/Google Hangouts)

Location (for in person interviews only):

eaTracker® app used: iOS Android version: __________

Device app is being used on (e.g., iPod, iPhone 5c, Samsung Galaxy s4): _________________________

Thank you very much for agreeing to participate in this study. The purpose of this project is to evaluate the eaTracker® mobile app. The information obtained from this work will help to understand user experiences with and perceptions of this app, and how to make it better. This study will also provide information to guide future modifications of the eaTracker® app and development of similar types of tools.

I am going to be asking you questions about your experiences with and thoughts about the eaTracker® mobile app and your thoughts about mobile apps in general for nutrition and physical activity behaviour change. This is a conversation more than anything and it should take up to 1 hour and there are no right and wrong answers. Is it ok if I audiotape the interview? This will allow me to accurately capture what was said. Again, I want to stress that everything that you say here is confidential and will only be heard by members of the research team. You won’t be identified personally. As well, if you are not comfortable answering any questions, please let me know and we can skip them. Likewise, if you do not want to continue the interview at any point, please let me know and we can stop. Do you have any questions before we begin?

What type of mobile device(s) (e.g., smartphone, tablet, iPod touch®) do you currently use?

Potential follow-up topics: Brand, model, operating system.

Have you used any other types of mobile devices in the past? If yes, what type of mobile device(s) did you use?

Approximately how long have you been using a mobile device? (both current and previous devices)

In general, what mobile apps do you currently use? Can you tell me how often you use mobile apps?

Potential follow-up topics: Has this changed over time?

Approximately how long have you been using mobile apps in general?

Can you tell me about your general day-to-day use of your mobile device and apps?

Potential follow-up topics: Has this changed over time?

Anything else you want to say about your overall use of mobile devices and apps?

How long have you been using the eaTracker® mobile app? (or how long did you use the eaTracker® mobile app?)

When was the last time you used the mobile app?

Typically how many times do you use the eaTracker® app in a week? in a day?

How do you decide to use the eaTracker® app? (do you have a schedule or a routine when you enter information or use the device?)

*We are now going to switch to talking about your healthy eating and physical activity goals in general and eaTracker in general.*

Can you please tell me about your healthy eating and/or physical activity goals? (what kinds of goals have you set for yourself or recorded on the eaTracker® device

Have you used the eaTracker® website in the past? **If yes**:

Can you please describe your use of the website (e.g., when did you first start using it? How often do you use it? ).

Can you please describe your general experiences with the eaTracker® website? What works (worked) well for you? What does (did) not work well for you?

*We are now going to switch to talk more specifically about the mobile apps to help you make diet and physical activity behaviour change and the eaTracker® app*

Can you describe your thoughts on mobile apps in general as a tool to help you make healthy eating and/or physical activity behaviour change?

Could you talk about the reasons behind your decision to download the eaTracker® mobile app?

Potential follow-up topics: When did you find out about the app? Could you tell me about how you found out about the eaTracker® mobile app? What initially attracted you to download this app?

When did you first download the eaTracker® mobile app? When did you last use the app? When actively using the app, how many days a week do you use it?

What were your thoughts about the app when you first downloaded it? (Did you find the app self-explanatory or did it take you come time to figure it out?)

Can you think of something that might have made the app easier to use in the beginning?

Do you find it easier or harder to initially use when compared to other mobile apps?

How many times did you have to use the app before you felt comfortable with it?

Did you ever get help using the app or website? (either from a healthcare professional, or EatRight Ontario, or help desk)?

If there were a function in the app to ask questions would you have used it?

With the eaTracker® app open, can you please walk me through your typical use of this app? I am interested in knowing your thoughts on different parts of the app.

**(Let participant walk you through first, and then ask about the first login, entering goals, tracking goals, deleting goals, entering food, entering physical activities, viewing My Messages etc).**

What has worked well for you with the eaTracker® app?

What has not worked well for you with the eaTracker® app?

Do you have any suggestions for improving the eaTracker® app?

Can you talk about what it was like to add the eaTracker® app into your normal life?

Could you please walk me through your day when using the eaTracker® app?

Potential follow-up topics: Frequency of use, locations of use, are apps used when users are around others? or alone?, are all data entered at once or throughout the day, when and where different features and components are used, day-to-day variability, change in usage over time, etc.

Can you talk about what it was like to use this app over and over again (or over time)?

Potential follow-up topics: What do you think would help you or others to successfully use this app over time? Were there times or situations when it was easier to use the app than others? Is there anything (e.g., feature) that would help to keep you motivated?

What are your thoughts on using the eaTracker® app in conjunction with the eaTracker® website?

(If Applicable) Can you please describe your experience using the eaTracker® app in conjunction with health professional assistance?

Potential follow-up topics: Type of professional visits where the eaTracker® app was used alongside care, was the eaTracker® app recommended to you by a health professional or did you initiate use on your own?, are the data from the app memory used during appointments and/or follow-up care?, were they supportive of app use? Were they knowledgeable about this app?

Have there been any benefits with using the eaTracker® app? If so, please describe. Have there been any drawbacks of using the eaTracker® app? If so, please describe.

Are you still using eaTracker® app? Can you tell me about the reasons that you are still using this app? Reasons that you are no longer using this app?

Do you expect to use the eaTracker® app in the future? Please explain.

Potential follow-up topics: Do you expect to mobile apps in the future for this purpose if they say no. Please explain.

Would you recommend the eaTracker® app to others? Please explain.

Is there another nutrition and/or physical activity app that you prefer to use over the eaTracker® mobile app?

If **yes,** what is the name of this mobile app?

What features about the other app do you like better than the eaTracker® app? (Ask them to show you this app and the features they like)

Can you please describe your progress in meeting your goals?

Potential follow-up topics: Do you think it would be different if you did not have access to the eaTracker app? Please explain.

Do you use any other tools/services to help you to set goals and/or to help you work towards achieving your healthy eating and physical activity goals? (can be mobile apps or something else)

If yes, what are these tools/services and can you tell me about your experiences.

If you could build a perfect mobile app to help people meet their healthy eating and/or physical activity goals, what would it look like?

Based on your experience with the eaTracker® app, is there anything that you want to say to:

1. Individuals thinking about using the eaTracker® app? Individuals currently using the eaTracker® app?
2. Developers looking to improve the eaTracker® app or develop other similar tools?

Of the following features, which would have appeal for you in a mobile app?

Look-up information (on calories or nutrients, restaurant menus etc.), general nutrition information/tips, goal setting, general motivational messages, motivational messages tailored to your specific information, opportunities to submit information to a dietitian and get feedback, chat rooms or forums with others trying to lose weight, chat room or forums with professionals, reminders (e.g., to complete food diaries), progress graphs, connections to social media to allow your data to be shared, social media pages related to the app, progress ‘competition’ against a buddy or group, notes section, water tracking tools, etc…. or is there anything else that you would like? not like?

Lastly we want to describe the age of our participants. Please classify your age into one of the following categories: 18-30y, 31-50y, 51-70y, 71+y

**Checklist**

Many of these points would have likely been covered during the interview. If there are any points that were not covered, participant thoughts on these topics and whether they affected app acceptance/use can be gathered at the end of the interview only.

-Was the app trialed/tested prior to use? Explain.

-Was the app easy or difficult to learn how to use? Explain.

-Amount of time it took to become comfortable with the app

-Thoughts on mental effort required to use the app

-Thoughts on how easy it was to remember how to use app

-Overall thoughts on app ease of use (e.g., understandable?, clear to use?, navigation?, easy to get it to do what they wanted it to do?)

-Can you describe whether other people (e.g., important people, health care professionals, family members, friends etc.) wanted you to use this app.

-Thoughts on app performance (e.g., how well did it perform tasks?)

-Thoughts on how applicable the app was to their situation

-Thoughts on app accuracy (including food and physical activity databases)

-Overall thoughts on usefulness to help facilitate the behaviour change process (e.g., allows things to be done more quickly?)

-Whether outcomes that others have had with the app can be seen? Did they talk to others about their experiences with the app prior to using it?

-Did they feel they had necessary skills and background to use the app?

-Thoughts on whether necessary resources were available to properly use the app (e.g., Internet connection, social/professional support, technical support etc.)

-Thoughts on app compatibility e.g., with their life, other apps, websites, etc.

**Interview Closure**

Is there anything else you would like to add?

I am very appreciative of the valuable information that you provided me today. It will go a long way in helping us to improve the eaTracker® mobile app. It will also help professionals, organizations, industry, and government to develop better mobile apps to help people positively change their eating and physical activity behaviours.

Thank you again for completing the interview. (Provide participant with Dietitians of Canada cookbook).

**Field notes:**

Sex: ______________

Rough weight classification: _______________

Did the participant appear to be tech savvy (explain):

_____________________________________________________________________________________

_____________________________________________________________________________________
